# The dementia and disability project in Thai elderly: rational, design, methodology and early results

**DOI:** 10.1186/1471-2377-13-3

**Published:** 2013-01-10

**Authors:** Vorapun Senanarong, Kamolthip Harnphadungkit, Niphon Poungvarin, Sathit Vannasaeng, Samut Chongwisal, Tipa Chakorn, Piyanuch Jamjumrus, Atthapon Raksthaput, Sinisa Chaichanettee, Nattapol Aoonkaew, Suthipol Udompunthurak, Rachelle S Doody, Jeffrey L Cummings

**Affiliations:** 1 Faculty of Medicine Siriraj Hospita, Mahidol University, Bangkok, Thailand; 2 Baylor College of Medicine, Texas, USA; 3 Lou Ruvo Center for Brain Health, Cleveland Clinic, 888 W Bonneville Ave, Las Vegas, NV, 89106, USA; 4 Division of Neurology, Department of Medicine, Faculty of Medicine Siriraj Hospital, Mahidol University, 2 Prannok Road, Bangkok, 10700, Thailand

**Keywords:** Mild cognitive impairment, Dementia, Alzheimer disease, Disability, White matter lesions, Thailand

## Abstract

**Background:**

A strong inverse relationship of functional limitation and socioeconomic status has been established in western ageing society. Functional limitation can be related to chronic diseases, disuse, cognitive decline, and ageing. Among chronic diseases in the Thai population, cerebrovascular diseases, diabetes, and arthritis are common. These factors are known to contribute to disability and poor quality of life in the elder population. Neuropsychiatric problems, cognitive decline, dementia, and cultural issues in elderly people also can alter the quality of life of the elderly.

**Methods:**

The Dementia and Disability Project in Thai Elderly (DDP) aims at comprehensively assessing community dwelling Thai elderly to understand the relationship between disability and motor function, neuropsychiatric symptoms, cognitive function, and chronic diseases. The DDP is the first study to look at the prevalence and etiology of dementia and of mild cognitive impairment (MCI) in Thai elders and to explore the relationship of cognition, disability, small vessel diseases and cortical degeneration with neuroimaging in Thai elderly people. 1998 Thai elders were screened in 2004–2006 and diagnosed as having MCI or dementia. 223 elders with MCI or dementia and cognitively normal elderly had brain magnetic resonance imaging (MRI) or at baseline. 319 elders from the 3 groups had blood tests to investigate the risks and possible etiologies of dementia including genotyping at baseline.

**Results:**

The mean age of elders in this study is 69.51(SD=6.71, min=60, max=95) years. 689(34.9%) are men and 1284(65.1%) are women. Mean body weight was 58.36(SD=11.20) kgs. The regression model reveals that performance on gait and balance and serum triglyceride predicts activity of daily living performance (adjusted r^2^ = 0.280, f=2.644, p=0.003). The majority of abnormal gait in Thai elders was lower level gait disturbance. Only 1.5% (29/1952) had highest level gait disorders. 39.5% of 1964 subjects were free of chronic diseases. Treatment gap (indicating those who have untreated or inadequate treatment) of diabetes mellitus and hypertension in Thai elders in this study was 37% and 55.5% respectively. 62.6% of Thai elders have ApoE3E3 allele. Prevalence of positive ApoE4 gene in this study is 22.85%. 38.6% of Thai elders who had MRI brain study have moderate to severe white matter lesions.

**Conclusion:**

The large and comprehensive set of measurements in DDP allows a wide-ranging explanation of the functional and clinical features to be investigated in relation to white matter lesions or cortical atrophy of the brain in Thai elderly population. An almost 2 year follow up was made available to those with MCI and dementia and some of the cognitively normal elderly. The longitudinal design will provide great understanding of the possible contributors to disability in the elderly and to the progression of cognitive decline in Thai elders.

## Background

Thailand has been an ageing society with older persons constituting more than 10 percent of the population since 2002. One of the important chronic diseases of Thai elderly population is dementia. The contributions of dementia and mild cognitive impairment (MCI) to disability in Thai elderly are unknown. Determining the causes of disability as well as the role of dementia and MCI in producing disability has major public health implications. Planning resource use, assisting families to cope and understanding reversible causes of disability are important application of this information. Deep cerebral small vessel disease caused by aging, arterial hypertension, diabetes mellitus, smoking and other factors is a vasculopathy leading to mobility problems and to disability [1,2]. Functional and clinical abnormalities in patients with cerebral small vessel disease, shown as white matter hyperintensities (WMHs) or white matter lesions (WMLs) in brain magnetic resonance imaging (MRI), have shown an association with global or selective cognitive deficits, depression, and motor abnormalities [3]. These features can contribute to disability in the elderly. Certain cognitive deficits, for example, slowing of processing time and executive dysfunction could give rise to difficulties in personal and social activities [4]. Motor deficits, including rigidity, gait apraxia and impaired balance on walking, increase risk of falls [5]. Physical illness such as back pain or osteoarthritis of the knees can also cause mobility problems. Disabilities in daily function and in general health are a possible consequence of an interaction between cognition, neuropsychiatric disorders, and chronic diseases in elderly persons.

### The dementia and disability project (DDP) on Thai elderly: rationale

An increase in interest in the burden of chronic and disabling health conditions that are not necessarily fatal has been growing in developing countries [6]. Few measures including the prevalence, disabilities and impairments related to the disorders, mortality and costs can indicate the full burden of such diseases [7]. In Thailand, there has been no epidemiology data on etiology of dementia or MCI in a community cohort. Understanding physical and mental health status of Thai elderly with and without disability will assist the formation of public health policy and response to the needs of social welfare for the elderly [8,9]. Longitudinal follow up can demonstrate the dynamic of these interactions. Epidemiological studies including brain imaging in Thai elderly can explain the natural history of the motor and mental disability from the biological point of view [10]. This may possibly lead to a preventive strategies based on scientific information.

This report aims to introduce the project and its background. This is a preliminary analysis of important baseline information and the associations between daily function and vascular risk factors from the Dementia and Disability Project (DDP). Future analyses on other issues from the DDP study are planned.

### Objectives

The specific aims of the DDP in Thai elderly are: 1) Determine the frequency of dementia, MCI and disability including Alzheimer disease (AD) as causes of disabilities in the Thai elderly community-dwelling cohort. 2) Determine the cause of dementia in elderly individuals. 3) Determine the incidence of dementia during a total follow-up period of 1–2 years on patients with MCI. 4) Determine the variance in disability of dementias contributed by behavioral disturbances and determine the relationship between functional disability, neuropsychiatric problems and quality of life in Thai elderly. 5) Explore the relationship between white matter lesions in brain imaging to functional autonomy disability, dementia, and depression in Thai elderly subjects. 6) Develop educational strategies or community service program for exporting this dementia and disability screening technique to other catchment areas in Bangkok or in other provinces.

## Methods

We screened Thai elders age 60 and above living in 42 villages in the catchment areas of the primary care unit (PCU) of Siriraj Hospital in late 2004-early 2006. The screening consisted of a comprehensive geriatric assessment to determine health status, functional status, cognitive function, mobility and balance, and possible disabilities. According to Persons with Disabilities Empowerment Act 2007 (B.E.2550) in Thailand, disability is classified into 5 domains: vision, hearing, daily function, neuropsychiatric issues, and cognition. We used a modified, culturally adapted Mini Mental State Examination (MMSE) called Thai Mental State Examination (TMSE) [11], Thai Activity of Daily Living (ADL) [12], Neuropsychiatric Inventory Questionnaire (NPI-Q) [13,14], Clinical dementia rating scale (CDR) [15], Thai Geriatric Depression Scale (TGDS) [16], Tinetti gait assessment [17], timed-get up and go test [18], standard neuropsychological tests [19-26] with culturally adjusted norms including tests of attention, memory, executive function, visuospatial function, and naming tasks. Hearing was assessed by standard audiometry. Profound hearing loss is described as being unable to hear a >90 deci Bell in the better ear. Visual acuity was assessed by using a pocket Jager chart. Severely impaired eyesight or blindness is defined by having corrected visual acuity <20/400 in both eyes. World Health Organization (WHO) quality of life was used to assess general health [27]. History of falls in the past 1 month was recorded. All is defined by a sudden, unintentional change in position causing an individual to land at a lower level, on an object, the floor, the ground or other surface and having part of the body touching the ground. We diagnosed dementia by Diagnostic and Statistical Manual (DSM) IV criteria [28] and MCI by modified Petersen’s criteria [29]. SPSS 11 was used for statistical analysis. Blood tests to analyze possible reversible causes of dementia and ApoE gene status, brain MRI were offered to those with MCI, dementia and to 30 cases of normal elders to evaluate the cause of dementia and its comorbidity.

The DDP planned to follow individuals with MCI and dementia at 1–2 year interval and to follow 5-10% of individuals with non-dementia non MCI (non-case group) for the same period. At the follow up assessment, the cognition, function, and neuropsychiatric problems were reassessed.

### Subjects

Sample size estimation based on detecting progression from MCI to demetia is calculated by assuming that prevalence of MCI is 15%, prevalence of MCI progression is 12%, 2-sided of 95% confidence interval and allowable error is 4%. Therefore, a sample size of MCI should be 254 and the studied population should be 1,694 (Computer program – n Query Advisor). If there is 15% drop out, then the sample size would be 1,948.

The DDP have been performed with the approval from the Ethics Committee on Research Involving Human Subject of Faculty of Medicine Siriraj Hospital, Mahidol University (No.141/2003). Written informed consent for participation in the study was obtained from both participants and their relatives either spouse or children.

### Inclusion

Inclusion criteria are as follows:

1. Elderly persons age 60 and above who register to obtain primary care service at the PCU of Siriraj Hospital.

2. Elderly persons and caregivers who agree to participate with the study for the 3 year period.

3. Elderly persons who live within 20 kilometers from Siriraj Hospital.

### Exclusion

Exclusion criteria are as follows:

1. Elderly persons or caregivers who do not wish to join the DDP study or are unable to give an informed consent.

2. Elderly persons who drop out because of the presence of severe illnesses (cardiac, hepatic or renal failure, cancer or other relevant systemic diseases) at the follow up, and who have severe illnesses at baseline and refuse to participate with the DDP study.

3. Severe psychiatric disorders.

### Baseline assessment

A structured and comprehensive questionnaire was administered by trained personnel to the patient and care partner and included information on demographic characteristics, education, occupational status defined as longest job in life, marital status, living conditions, lifestyle habits (alcohol consumption, smoking), vascular risk factors and current medical problems. Elders and caregivers were purposely asked if elders had any memory complaint or similar problem. Blindness and severe hearing loss were determined with the assessment.

### Functional and clinical assessments

Mobility is assessed by timed get up and go test (TUG) and Tinetti gait assessment score. TUG starts with an individual rising from a chair, walking 3 meters, turning around, returning to the chair, and siting down. TUG test measures the time taken to complete the test. One practice trial is given before the test. Results of <10 seconds indicates freely mobile, <20 seconds - mostly independent, 20–29 seconds - variable mobility, and >20 seconds suggests impaired mobility. The grading of get up and go (GUG) [30] is modified into 5 grades in which 1 is normal or no fall risk and 5 is severely abnormal or very high fall risk. The Tinetti balance and gait evaluation is divided in two parts: the first part studies balance defects based on 9 postural situations and the second part studies gait. The maximum score is 28 in which a higher score indicates a better balance and gait performance. Activities of daily living (ADLs) are assessed by utilizing the Thai ADL scale which consists of 6 basic ADLs and 7 instrumental ADLs and scored as 01, 2. A higher score of Thai ADLs indicates poorer daily function.

The NPI-Q assesses 12 neuropsychiatric including mood, psychotic, vegetative, and frontal related symptoms or behaviors. It starts with asking if the symptom is present (1= yes, 0= no) and asking about the severity of the symptoms (1= mild, 2= moderate, 3=severe). The NPI includes additional frequency assessment that scoring 1 to 4 (1 = occasionally, less than once per week; 4 = very frequently, once or more per day or continuously). The NPI-Q was used at the screening phase at baseline and the NPI was used in those who came into the second phase of the study to assess their dementia and MCI status. Caregivers or family members familiar with the individual’s behavior are interviewed using scripted questions. The caregiver is asked if the individual’s behavior has changed during the past month. Thai GDS consists of 30 questions; a higher score signifies more depressive symptoms. Score 0–12 implies no depression, score 13–18 suggests mild depression, score 19–20 indicates moderate depression, and score 25–30 denotes severe depression. An individual is asked to consider if the symptoms are present in the past 1 week. The CDR is a global assessment instrument of dementia that yields global and sum of boxes (SB) scores, with the global score being indicative of stage dementia severity while CDR-SB being considered a more detailed quantitative general index than the global score [31]. The CDR assesses 6 domains of functioning: memory, orientation, judgment and problem solving, community affairs, home and hobbies, and personal care. Each domain is rated on a 5-point scale of functioning as follows: 0, no impairment; 0.5, questionable impairment; 1, mild impairment; 2, moderate impairment; and 3, severe impairment (personal care is scored on a 4-point scale without a 0.5 rating available).

TMSE is a screening tool for cognitive impairment. Those with TMSE ≤ 26 were recruited to evaluate cognitive status. 5-10% of cognitively normal elderly were examined (74 cognitively normal elderly, 5.05%). The DDP is also interested in the association of cognition and other chronic diseases. Comorbid diseases that are included in this study are hypertension (BP≥140/90 mmHg or on antihypertensive drugs); pulmonary diseases (such as chronic obstructive pulmonary disease: having history of chronic progressive symptoms namely cough and/or wheeze and/or breathlessness with objective evidence of airways obstruction that does not return to normal with treatment, taking medication to help ventilation/breathing problems, asthma, and restrictive lung diseases); heart disease (coronary heart disease, valvular heart disease, and heart failure); thyroid diseases (hypothyroidism, hyperthyroidism, or taking medication for thyroid); diabetes mellitus (taking hypoglycemic drugs, taking regular insulin injection, or fast blood sugar ≥126 mg%); arthritis (history of or on examination present osteoarthritis (OA). (OA of the knee is characterized by knee pain for most days of the prior month, OA of the hand is characterized by hand pain, aching or stiffness for most days of the prior month, OA of the hip with hip pain for most days of the prior month); crystal induced arthritis, and rheumatoid arthritis); cerebrovascular disease (taking platelet anti aggregation drugs and a history of previous stroke, having a history of stroke, or hemiparesis on neurological examination); smoker (current or active smoking); and alcohol drinker (current or active alcohol drinking). The World Health Organization (WHO) defines health as a state of complete physical, mental, and social well-being, not merely the absence of disease and has invented a measurement of the improvement in the quality of life related to health care. WHO describes Quality of Life (QoL) [27]as an individual's perception of their position in life in the context of the culture and value systems in which they live and in relation to their goals. The WHOQOL-BREF in this study is based on a four domain structure: physical health, psychological health, social relationships, and environment. The WHOQOL-BREF is self-administered to elderly individuals with family members or caregivers as assistants. Standardized instructions were read to respondents when the assessment is interviewer-administered. The WHOQOL-BREF initial raw scores are converting to transformed scores with the second transformation converting domain scores to a 0–100 scale. The initial rating score ranges from 1 (very dissatisfied, never, not at all, very poor) to 5 (very satisfied, always, extremely, very good).

### Magnetic resonance imaging study

All MCI, dementia, and some cognitively normal elderly subjects were offered a brain MRI study within 6 months after the diagnosis of dementia or MCI was made. A total of 223 subjects had brain MRI. The MRI scan was done with 1.5T. (Intera, Philips, the Netherlands) field strength. The protocol composed of 3D-T1wi in coronal plane perpendicular to temporal lobe axis (iso-voxel 1×1×1 mm), axial FLAIR and T2wi (thickness 5 mm). The 3D-T1wi was reformatted to axial plane and used for volumetric measurement of hippocampus. Axial gradient echo T2wi was performed in some cases. Visual rating scale of medial temporal lobe area (MTA) [32], Fazekas WML [33], and Scheltens WML rating scale (SS) [34] were utilized in brain MRI reading by radiologist (O. Chawalparit, OC) and neurologist (V. Senanarong, VS). Volumetric study by region of interest (ROI) technique was done by OC and VS. VS and OC obtained rater standardization evaluation with a weighted kappa variance score <10%. The WMLs were visually rated on the axial FLAIR image with the use of two semiquantitative rating scales. The Fazekas scale is a four-point scale (none, mild, moderate, severe) rating the extent and severity of the deep white matter changes and the Scheltens scale, in which WMLs are rated 0–6 in 13 subcortical regions, including the basal ganglia and infratentorial region, and 0–2 in 3 periventricular regions, resulting in a total range of 0–84. The global cortical atrophy (GCA) [35] was rated on the axial FLAIR image using a 4-point visual rating scale (0 = none, 1 = widening of sulci, 2 = atrophy of gyri, 3= end stage atrophy with ‘razor-blade’ gyri). The MTA was visually rated on 3D-T1wi in coronal plane using a 5-point scale (0 = none, 1 = widening of Choroid fissure, 2 = widening temporal horn, opening fusiform and collateral sulcus, 3 = profound volume loss hippocampus, 4 = end stage atrophy).

### Follow-up assessment

Clinical data on daily function, cognition, and neuropsychiatric symptoms were reassessed in MCI, dementia and cognitive normal elderly (30 cases in this study) at a 1–2 year interval. In this study the mean follow up time on 288 subjects is 529.96 days (SD=230.73 days, median 433 days, min 272 days, max 1,312 days). Evolution of cognitive function especially to a diagnosis of dementia was assessed at the follow up, by a consensus group (neurologists, psychologists, radiologist and physicists).

### Statistical analysis

Statistical analysis was performed using SPSS 11 (SPSS Inc., Chicago, IL) We examined baseline demographics, risk factors, and comorbid features by assessing significance by means of Chi square test for categorical variables. We assessed the association between activities of daily living and vascular risk factors or relevant predictors with Pearson or Spearman correlation coefficient of the whole data and then performed linear regression analysis. We assessed the association between WML, cognitive function and general brain atrophy with Pearson correlation coefficient. The subjects with MCI or dementia and cognitively normal elderly were compared across demographic characteristics (age, sex, education, living conditions, employment status) using t tests. Risk factors of cognitive decline were compared using one-way ANOVA with Bonferoni adjustment or Chi square test. Epi info 6 was used to find 95% confidence interval of the prevalence and odds ratio. P<0.05 is considered to be of statistical significance.

## Results

**Figure 1:**
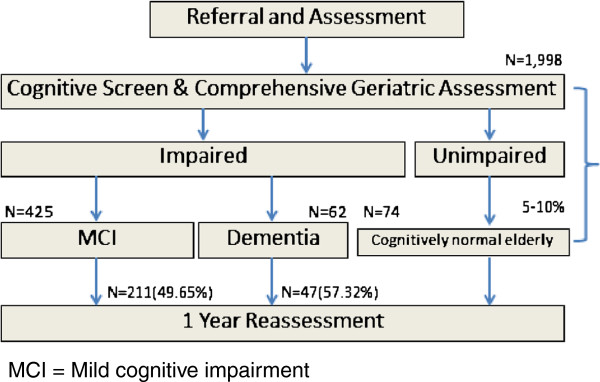
**Diagnoses of the dementia and disability project (DDP) cohort from the period of 2004–2006.** MCI = Mild cognitive impairment.

One thousand nine hundreds and ninety-nine elders were screened. Only 1973 subjects had adequate data for statistical analysis. The DDP study summary is shown in Figure 1 and subject baseline characteristics are shown in Table 1. 951(48.2%) elders had memory complaints (considered by either elders or/and caregivers). The GUG test reveals that 1442 elders (76.9%) had no fall risk, 225(12%) had low fall risk, 116(6.2%) had some fall risk, 73(3.9%) had high fall risk, and 18(1%) had very high fall risk. However, two hundred and one elders (201/1962=10.24%) reported having falls in the previous 1 month. Regarding Thai ADL assessment scale, 75% of these Thai elders are independent in IADL, 10.4% had impairment in 1 IADL, and 14.6% had 2 or more IADL impairments. The majority of these elders (93.3%) is independent for basic ADL. 3.9% and 1.2% had 1 and 2 impairment in basic ADL. Prevalence of dependency in basic and instrumental ADLs is shown in Table 2. Almost 4% of this elderly cohort reported having urinal or fecal incontinence. Nearly 20% of this elders reported partial or total dependence on travelling or utilizing public transportation or commuting into a community. Walking assessment [36] also reveals that the prevalence of lower level gait disturbance is 20% (395/1952), that of middle level gait disturbance is 2.6% (52/1952), that of higher level gait disturbance is 1.5% (29/1952), and the prevalence of normal gait is 74.8%(1476/1952). In Table 3, we found fair to moderately good associations (r=0.25 to r=0.75) between function (activities of daily living) and age, weight, TMSE, balance and gait measures (Tinetti balance & gait assessment, TUG). Low correlations (r<0.25) between function and depression (Thai GDS), systolic blood pressure, gender, previous occupation, levels of income, blood levels of homocysteine, history of taking hypnotics, history of having thyroid disorders or previous stroke or osteoarthritis/rheumatoid arthritis or heart disease or diabetes mellitus or hypertension, and alcohol drinking were discovered. All significant associations are lost when controlled with age. A linear regression model to predict performance of activities of daily living reveals that adjusted R^2^ is 0.280 (F=2.644, p=0.003), and that Y= 0.214+0.32(TUG) + 0.25(triglyceride levels). Table 4 and 5 shows treatment gap of DM and hypertension. Simple definition of the treatment gap is the number of people with a condition or disease who need treatment for it but who do not get it or who receive inadequate treatment. We found that 37% of those elders with abnormal Fasting Blood Sugar (FBS) either did not have hypoglycemic treatment or knew that they had DM but did not take medication [Chi square p= 0.044, df = 2 (6.229)] and 63%[(560+669)/1951] of elders had either under diagnosed hypertension or inadequate treatment of hypertension [Chi Square p<0.001, df=1(68.075)]. Table 6 shows frequency of comorbidity in Thai elders. Only 781 (39.5% of 1964 subjects) were free of comorbid diseases (e.g. hypertension, pulmonary diseases, heart diseases, thyroid diseases, DM, arthritis, cerebrovascular disease, smoker, alcoholic drinker). 668 (34%) had 1 chronic disorder, 381 (19.4%) had 2 comorbid diseases, 102 (5.2%) had 3 comorbid diseases, 28 (1.4%) had 4, 3 (0.2%) had 5, and 1 (0.1%) had 6 comorbid diseases. Table 7 shows ApoE gene status. 22.85% of this elderly cohort (N=302) are ApoE4 positive. Table 8 demonstrated the result of brain MRI WMLs. Ninety percent of Thai community dwelling elders have WMLs. Of those 90%, 52% have mild WMLs and 38.6% have moderate to severe white matter lesions.

**Table 1:** Subject baseline characteristics and their blood test results

**Character (N=1973)**	**mean(SD) or N(%)**
Age (years)	69.51(6.71)
Weight (Kgms)	58.36(11.20)
TMSE	26.06(3.62)
Thai ADL scale scores	0.83(2.35)
Tinetti balance & gait assessment (N=868)	23.72(2.92)
Tinetti balance subscale assessment (N=868)	15.25(1.81)
Tinetti gait subscale assessment (N=868)	8.47(1.28)
Sum Thai GDS	9.35(6.65)
Serum B12 (pmol/L) (N=309)	448.85(229.43)
Serum folate (nmol/L) (N=309)	7.83(5.29)
Serum homocystein (μmol/L) (N=309)	14.42(5.13)
Serum total cholesterol (mg/dL) (N=318)	223.56(48.23)
Fasting blood sugar (mg/dL) (N=318)	113.41(47.36)
Serum triglyceride (mg/dL) (N=318)	157.09(96.76)
Hemoglobin (gm/dL) (N=319)	12.79(1.89)
Blood pressure (mmHg)	140.46(20)/81.64(10.47)
Timed get up and go (TUG) (seconds)	13.22(7.27)
Gender: male/female	689(34.9)/1284(65.1)
Religion: Buddhist/Islam/Christian/ Other (N=1962)	1907(97.2)/51(2.6)/2(0.1)/2(0.1)
Marital status: married/ single/ widowed or divorced (N=1960)	1009(51.5)/ 146(7.4)/ 805(41.1)
Levels of education: no formal years of education/ < 4 years/4 years/ >4-6 years/ >6-9 years/Diploma levels/ >=bachelor degree (N=1968)	224(11.4)/ 273(13.9)/ 934(47.5)/ 167(8.5)/152(7.7)/137(7)/ 81(4.1)
Previous occupation: Civil Servant/Business/ employee/ agriculturist/ Other (N=1967)	369(18.8)/ 480(24.4)/ 415(21.1)/ 220(11.2)/ 483(24.6)
Currently still working (N=1966)	1043(53.1)
Levels of income: very poor / not enough or poor/ sometimes insufficient/sufficient income/good income with some saving (N=1938)	60(3.1)/403(20.8)/490(25.3)/809(41.7)/176(9.1)
Living status : alone/ with family/ with non-family/ other (N=1964)	125(6.4)/ 1752(89.2)/ 27(1.4)/ 60(3.1)

TMSE = Thai Mini Mental State Examination ADL = Activity of daily living.

GDS = Geriatric depression scale.

**Table 2:** Prevalence of dependence on activity of daily living in Thai elderly

**N=1956**	**Independent(%)**	**Partial support/ need supervision(%)**	**Total dependence(%)**
Bathing	1935(98.9)	12(0.6)	9(0.5)
Dressing	1938(99.1)	12(0.6)	6(0.3)
Toileting	1943(99.1)	6(0.3)	7(0.4)
Transferring	1922(98.3)	26(1.3)	8(0.4)
Bowel/ bladder incontinence	1885(96.4)	58(3) occasionally or 1–2 times per week	13(0.7) a few times per week
Eating	1936(98.9)	17(0.9)	3(0.2)
Ability to use a telephone	1732(88.5)	144(7.4)	80(4.1)
Travelling or utilizing public transportation or commuting into a community	1596(81.6)	291(14.9)	69(3.5)
Grocery/housewhole shopping	1823(93.2)	86(4.4)	47(2.4)
Cooking	1839(94)	71(3.6)	46(2.3)
Doing housework	1864(95.3)	62(3.2)	30(1.5)
Ability to take her/his own medication	1875(95.9)	57(2.9)	24(1.2)
Money handling	1862(95.2)	68(3.5)	26(1.3)

**Table 3:** Correlations between ADL (Thai ADL scale scores) and vascular risk factors or relevant factors

**Character (N=1973)**	**mean(SD) or N(%)**	**Pearson correlation**
	0.83(2.35)	Thai ADL scale scores
Age (years)	69.51(6.71)	0.305(**)
Weight (kgs)	58.36(11.20)	−0.110(**)
TMSE	26.06(3.62)	−0.505(**)
Tinetti balance & gait assessment (N=868)	23.72(2.92)	−0.344(**)
Tinetti balance subscale assessment (N=868)	15.25(1.81)	−0.360(**)
Tinetti gait subscale assessment (N=868)	8.47(1.28)	−0.303(**)
Sum Thai GDS	9.35(6.65)	0.139(**)
Serum B12 (pmol/L) (N=309)	448.85(229.43)	−0.074
Serum folate (nmol/L) (N=309)	7.83(5.29)	−0.066
Serum homocystein (μmol/L) (N=309)	14.42(5.13)	0.152(**)
Serum total cholesterol (mg/dL) (N=318)	223.56(48.23)	−0.020
Serum triglyceride (mg/dL) (N=318)	157.09(96.76)	−0.007
Fasting blood sugar (mg/dL) (N=318)	113.41(47.36)	−0.049
Hemoglobin (gm/dL) (N=319)	12.79(1.89)	−0.025
Blood urea nitrogen (mg/dL)(N=317)	15.36(5.44)	−0.013
Serum creatinine (mg/dL) (N=319)	0.9235(0.54)	0.019
Blood pressure (mmHg)	140.46(20)/81.64(10.47)	0.054(*)/0.032
Timed get up and go (TUG) (seconds)	13.22(7.27)	0.397(**)
Gender: male/female	689(34.9)/1284(65.1)	0.173(**)(Spearman correlation)
Previous occupation: Civil Servant/ Business/ employee/ agriculturist/ Other (N=1967)	369(18.8)/ 480(24.4)/ 415(21.1)/ 220(11.2)/ 483(24.6)	0.102(**)(Spearman correlation)
Levels of income: very poor / not enough or poor/ sometimes insufficient/ sufficient income/ good income with some saving (N=1938)	60(3.1)/403(20.8)/490(25.3)/809(41.7)/176(9.1)	−0.157(**)(Spearman correlation)
Living status: alone/ with family/ with non-family/ other (N=1964)	125(6.4)/ 1752(89.2)/ 27(1.4)/ 60(3.1)	0.043(Spearman Correlation)
History of smoking: yes/ex smoking/no (N=1967)	242(12.3)/379(19.3)/1346(68.4)	0.040(Spearman Correlation)
History of alcohol drinking: yes/ex drinking/no (N=1961)	193(9.8)/483(24.6)/1285(65.5)	(0.046(*)(Spearman Correlation)
History of hypertension: yes/no (N=1951)	753(38.6)/ 1198(61.4)	−0.100(**)(Spearman Correlation)
History of chronic obstructive pulmonary disease: yes/no (N=1964)	71(3.6)/ 1893(96.4)	−0.024(Spearman Correlation)
History of heart disease: yes/no (N=1962)	216(11.0)/ 1746(89.0)	−0.098(**)(Spearman Correlation)
History of diabetes mellitus: yes/no (N=1964)	330(16.8)/ 1634(83.2)	−0.070(**)(Spearman Correlation)
History of rheumatoid arthritis or osteoarthritis: yes/no (N=1962)	379(19.3)/ 1583(80.7)	−0.087(**)(Spearman Correlation)
History of stroke: yes/no (N=1962)	51(2.6)/ 1911(97.4)	−0.124(**)(Spearman Correlation)
History of any thyroid disorders: yes/no (N=1962)	63(3.2)/ 1899(96.8)	0.048(*)(Spearman Correlation)
History of any comorbid diseases: yes/no (N=1964)	1183(60.2)/ 781(39.8)	0.131(**)(Spearman Correlation)
History of taking hypnotics or anxiolytics: yes/no (N=1958)	251(12.8)/ 1707(87.2)	−0.063(**)(Spearman Correlation)
History of taking antidepressants: yes/no (n=1952)	11(0.6)/1941(99.4)	−0.026(Spearman Correlation)
History of taking antipsychotics: yes/no (N=1952)	7(0.4)/1945(99.6)	0.011(Spearman Correlation)

* = p<0.05 ** = p<0.01.

ADL= Activity of daily living, TMSE= Thai Mini Mental State Exam, GDS = Geriatric depression scale.

**Table 4:** Participants who have untreated diabetes mellitus or hypertension

**History of DM**		**FBS <100 mg%**	**FBS 100-125 mg%**	**FBS >125 mg%**	**Total**
Yes	Take medication	6 (17.1%)	5 (14.3%)	**24 (68.6%)**	35 (100%)
No	No	2 (25%)	4 (50%)	**2 (25%)**	8 (100%)
	No	165 (62%)	69 (25.9%)	**326 (12%)**	266 (100%)

Chi square p= 0.044*.

df = 2 (6.229).

Participants who have untreated diabetes mellitus are 37% (25%+12%=37%) of those elders with abnormal FBS.

**Table 5:** Participants who have a history of hypertension and actually have taken antihypertensive drugs

		**Normal or well controlled BP (<140/90)**	**High BP (≥140/90)**	**Total**
History of hypertension (divided into those with adequate treatment with good BP controlled (<140/90) and inadequate treatment with poor BP controlled (BP>140/90)	Yes	193 (25.6% of those on antihypertensive drugs & having well controlled BP) 529 (44.2%)	**560 (74.4% of those on antihypertensive drugs but having poor controlled BP)**	753 (100%)
	No		669 (55.8% of those without a history of hypertension but having high BP)	1198 (100%)
	Total	722 (37%)	1229 (63%)	1951 (100%)

Chi Square p<0.001.

df = 1(68.075).

Thai elders who have untreated hypertension or have inadequate treatment of hypertension (HT) are 63% [(560+669)/1951=62.99%].

**Table 6:** Comorbidity in Thai elderly cohort

		**Number of comorbidities**						**Total**	
		**0(n, % in row, % in column)**	**1(n, % in row, % in column)**	**2(n, % in row, % in column)**	**3(n, % in row, % in column)**	**4(n, % in row, % in column)**	**5(n, % in row, % in column)**	**6(n, % in row, % in column)**	**(n, % in row, % in column)**
Age group	60-64	219(41.56, 30.81)	175(33.21, 26.16)	92(17.46, 22.49)	31(5.88, 24.22)	9(1.71, 23.08)	0	1(0.19, 100)	527(100, 26.86)
	65-69	202(35.88, 28.41)	205(36.41,30.64)	114(20.25, 27.87)	30(5.33, 23.44)	12(2.13, 30.77)	0	0	563(100, 28.70)
	70-74	141(32.64, 19.83)	146(33.80,21.82)	100(23.15, 24.45)	30(6.94, 23.44)	12(2.78, 30.77)	3(0.69, 60)	0	432(00, 22.02)
	75-79	88(32.23, 12.38)	89(32.60, 13.30)	62(22.71, 15.16)	27(9.89, 21.09)	5(1.83, 12.82)	2(0.73, 40)	0	273(100, 13.91)
	80+	61(36.53, 8.58)	54(32.34, 8.07)	41(24.55, 10.02)	10(5.99, 7.81)	1(0.6, 2.56)	0	0	167(100, 8.61)
Total		711(36.24, 100)	669(34.10,100)	409(20.85, 100)	128(6.52, 100)	39(1.99, 100)	5(0.25, 100)	1(0.05, 100)	1962(100, 100)

[Pearson Chi square p=0.064, df=24 (35.313)].

**Table 7:** ApoE gene status

**ApoE gene (N=302)**	**Frequency**	**%**
E2E2	2	0.7
E2E3	42	13.9
E2E4	11	3.6
E3E3	189	62.6
E3E4	55	18.2
E4E4	3	1.0

**Table 8:** Fazekas score of brain MRI (N=223)

**Fazekas grade**	**Frequency**	**%**
0	21	9.4
1	116	52.0
2	60	26.9
3	26	11.7

## Discussion

This DDP study is the first attempt in the Thai elderly population to assess the prevalence of cognitive problems in association with disability and to follow individuals with cognitive problem. In this study, a linear regression adjusting for covariates showed that only gait and balance and triglycerides were significantly associated with activities of daily living after adjusting for the other variables. Other variables that were significant before adjustment and fell out of the final model include advanced age, gender, cognitive function, depressive mood, weight, systolic blood pressure, previous occupation, levels of income, blood levels of homocysteine, history of taking hypnotics, history of having thyroid disorders or previous stroke or osteoarthritis/rheumatoid arthritis or heart disease or diabetes mellitus or hypertension, and alcohol drinking. Our elderly are rather mobile. 75% are independent in IADLs and 93.3% are independent in basic ADLs. Previous studies also showed that advance in age, presence of diabetes, stroke, and depressive symptomatology were independent features associated with physical disability. WMLs have been reported to be associated with selective cognitive function, mobility, mood, and the loss of independence in old age populations. In an epidemiological study of Nigerian elderly population, it was found that, according to the six-item ADL index, the prevalence of physical disability for people aged 65 was 21.4% [37]. Sixty three percent of the Thai cohort has at least one chronic disease as a comorbidity of dementia. Most have 1–2 chronic diseases. Only six percent of Thai elders in this study live alone. These are important points in terms of public planning to reassure planners of the magnitude and value of family members as care-givers. Vascular risk factors contribute to small vessel diseases and dementia. In our study, 38.6% of Thai elders who had MRIs have moderate to severe white matter lesions. These findings indicate the high prevalence of small vessel diseases on our Thai cohort. Our study reveals a treatment gap (indicating participants who have untreated or inadequate treatment) of DM and hypertension in elderly subject. The treatment gap of chronic diseases, especially those related to vascular risks is an important issue for disease prevention. To-date, there is no disease modifying drug for dementia. A recent proposal [38] to look at seven modifiable risk factors for AD (diabetes, midlife hypertension, midlife obesity, smoking, depression, cognitive inactivity or low educational attainment) stated that half of AD cases worldwide are potentially attributable to these risk factors. A 10 to 25% reduction in these risk factors could potentially lead to a reduction of 1.3 to 3 million AD cases. Existence of vascular risk factors and chronic diseases can lead to falls, osteoporosis, poor mental health and poor mobility outcome. From the LADIS study [39], elders with WMLs had cognitive complaint, psychic complaint, and previous minor stroke. More motor complaint was found in those with more WMLs on brain imaging. Our study reaffirms that Thai elderly population has highest allele frequency of ApoE 3 gene, which is consistence with previous study [40]. The frequency of ApoE4 gene is 22.85% in the current study.

Assessment of gait in the elderly is as important as functional assessment. The majority of Thai elders has normal gait and the second common cause of abnormal gait is low level gait disorder. The basic neural control of walking on 2 legs consists of equilibrium, locomotion, and other non- neurologic factors (mechanical support system and general health). Abnormal gait syndromes [36] can be divided into lower level gait disorder (peripheral skeletomuscle problems and peripheral sensory problems), middle level gait disorder (hemiplegic gait, paraplegic gait, cerebellar ataxic gait, Parkinsonian gait, choreic gait, and dystonic gait), and highest level gait disorder (cautious gait, subcortical disequilibrium, frontal disequilibrium, isolated gait ignition failure, and frontal gait disorder). Furthermore, neurologic abnormalities affecting gait occur early in several types of non-Alzheimer's dementias. Verghese J, et al. found that subjects with neurologic gait abnormalities had a greater risk of development of dementia (hazard ratio, 1.96 [95 percent confidence interval, 1.30 to 2.96]) [41]. Among the types of abnormal gait, unsteady gait predicted vascular dementia (hazard ratio, 2.61), as did frontal gait (hazard ratio, 4.32) and hemiparetic gait (hazard ratio, 13.13). The importance of deterioration in the control of balance as a reason leading to abnormal gait in the elderly is well recognized. Assessment of gait problems can be part of fall prevention and dementia evaluation. Additionally, one contributor of abnormal gait is a musculoskeletal issue that is less flexible and decreasing in strength. Poor motor plan in elderly individuals is another factor. Thus, we can signify a relationship between comorbidity and disability.

Once again, this is an initial study on imperative baseline information and the relations between daily function and vascular risk factors. Future analysis of the data will be carried out on other issues. The limitation of this study is that it includes only elderly living in Bangkok Metropolitan areas. It may not represent the whole country but it is the first study in Thailand to look at dementia, MCI and chronic disorders. Our investigation on prevalence of white matter lesions in community dwelling Thai elders is the first epidemiologic neuroimaging study in Thailand.

## Conclusions

The DDP study offers the possibility of exploring the role of dementia and functional disabilities in relation to the evolution of cognitive decline in an ageing Thai population. The DDP comprises a large and comprehensive test battery and explores global and selective functions of cognition, mobility, and daily activity. An epidemiological study of brain MRI is conducted for the first time in Thai population in this study.

## Competing interest

The authors declare that we have no competing interests with this study.

## Authors’ contributions

Vorapun Senanarong, JL Cummings: Research planning, primary investigators of the awarding grants, conducting the field study, data analysis and interpretation of the results, preparing manuscript. Kamolthip Harnphadungkit, Niphon Poungvarin, Sathit Vannasaeng, Rachelle S Doody: Assisting research planning, assisting data analysis and interpretation of the results. Samut Chongwisal, Tipa Chakorn, Piyanuch Jamjumrus: Conducting field study. Athapol Raksapad, Sinisa Chaichanetree, Nattapol Aoonkaew: Conducting field study, assessment of cognition and neuropsychiatric problems, preparing the data for statistical analysis. Suthipol Udompunthurak: Assisting research planning, data manager, statistical analysis. All authors read and approved the final manuscript.

## Pre-publication history

The pre-publication history for this paper can be accessed here:

http://www.biomedcentral.com/1471-2377/13/3/prepub

## References

[B1] Longstreth WT Manolio TA Arnold A Burke GL Bryan N Jungreis CA Enright PL O’Leary D Fried: clinical correlates of whitematter findings on cranial magnetic resonance imaging of 3,302 elderly people. The Cardiovascular Health Study Stroke 1996 27 1274 1282 10.1161/01.STR.27.8.1274 8711786

[B2] Inzitari D Pracucci G Poggesi A Carlucci G Barkhof F Chabriat H Changes in white matter as determinant of global functional decline in older independent outpatients: three year follow up of LADIS (leukoaraiosis and disability) study cohort BMJ 2009 339 b2477 online first 1–9 10.1136/bmj.b2477 19581317 PMC2714680

[B3] Teodorczuk A O'brien JT Firbank MJ Pantoni L Poggesi A Erkinjuntti T White matter changes and late-life depressive symptoms: longitudinal study BJP 2007 191 212 217 10.1192/bjp.bp.107.036756 17766760

[B4] Senanarong V Poungvarin N Jamjumras P Sriboonroung A Danchaivijit C Udomphanthuruk S Cummings JL Neuropsychiatric symptoms, functional impairment and executive ability in Thai patients with Alzheimer's disease Int Psychogeriatr 2005 17 1 81 90 10.1017/S1041610205000980 15945593

[B5] Baloh RW Ying SH Jacobson KM A longitudinal study of gait and balance dysfunction in normal older people Arch Neurol 2003 60 835 839 10.1001/archneur.60.6.835 12810488

[B6] Beaglehole R Yach D Globalisation and the prevention and control of non‐communicable disease: the neglected chronic diseases of adults Lancet 2003 362 903 908 10.1016/S0140-6736(03)14335-8 13678979

[B7] Mathers CD Loncar D Projections of global mortality and burden of disease from 2002 to 2030 PLoS Med 2006 3 11 e442 Available at: http://www.plosmedicine.org/article/info:doi/10.1371/journal.pmed.0030442. Accessed February 21, 2009 10.1371/journal.pmed.0030442 17132052 PMC1664601

[B8] Eaton WW Martins SS Nestadt G Bienvenu OJ Clarke D Alexandre P The burden of mental disorders Epidemiol Rev 2008 30 1 14 10.1093/epirev/mxn011 18806255 PMC2683377

[B9] Brookmeyer R Johnson E Ziegler-Graham K Michael Arrighi H Forecasting the global burden of Alzheimer’s disease Alzheimers Dement 2007 3 186 191 10.1016/j.jalz.2007.04.381 19595937

[B10] Inzitari D Simoni M Pracucci G Poggesi A Maria Basile A Chabriat H Risk of rapid global functional decline in elderly patients with severe cerebral age-related white matter changes. The LADIS study Arch Intern Med 2007 167 81 88 10.1001/archinte.167.1.81 17210882

[B11] Train the brain forum committee Thai Mental State Examination (TMSE) Siriraj Hosp Gaz 1993 45 359 374

[B12] Senanarong V Harnphadungkit K Prayoonwiwat N Poungvarin N Sivasaruyanonb N Printarakul T A new measurement of activities of daily living for Thai elderly with dementia Int Psychogeriatr 2003 15 135 148 10.1017/S1041610203008822 14620072

[B13] Cummings JL Mega M Gray K The Neuropsychiatric Inventory: comprehensive assessment of psychopathology in dementia Neurology 1994 44 2308 2314 10.1212/WNL.44.12.2308 7991117

[B14] Kaufer DI Cummings JL Ketchel P Smith V MacMillan A Shelley T Validation of the NPI-Q, a brief clinical form of the Neuropsychiatric Inventory J Neuropsychiatry Clin Neurosci 2000 12 233 239 10.1176/appi.neuropsych.12.2.233 11001602

[B15] Morris JC The Clinical Dementia Rating (CDR): current version and scoring rules Neurology 1993 43 2412 2414 8232972 10.1212/wnl.43.11.2412-a

[B16] Train the Brain Forum Committee Thai Geriatric Depression Scale. TGDS Siriraj Hosp Gaz 1994 46 1 9

[B17] Tinetti ME Performance oriented assessment of mobility problems in elderly patients J Am Geriatr Soc 1986 34 119 126 3944402 10.1111/j.1532-5415.1986.tb05480.x

[B18] Podsiadlo D Richardson S The time “Get Up and Go” test: a test of basic functional mobility for frail elderly persons J Am Geriatr Soc 1991 39 142 148 1991946 10.1111/j.1532-5415.1991.tb01616.x

[B19] Weschsler D WMS-R Wechsler Memory Scale- Revised Manual 1987 The Psychological Corporation, Harcourt Brace Jovanovich, Inc, New York

[B20] Seeboonruang A The comparative study about anomia between demented elderly and normal ageing by Boston naming test. Thesis from master degree of science(clinical psychology) 2003 Faculty of Graduate Studies, Mahidol University, Bangkok ISBN 974-044-3747-8

[B21] Srisarakorn P The study of Wechsler memory scale 3rd edition in the elderly demented patents. Thesis from master degree of science programme in clinical psychology Faculty of Medicine Siriraj Hospital. Mahidol University

[B22] Opasanon N A study of the Wechsler memory scale 3rd edition abbreviated in the elderly dementia patients. Thesis from master of science program in clinical psychology Faculty of Medicine Siriraj Hospital. Mahidol University

[B23] Lu L Bigler ED Normative data on trail making test for neurologically normal Chinese-speaking adults. Appl Neuropsychol 2002 9 219 225 10.1207/S15324826AN0904_4 12665458

[B24] Chompuchum P Wongsepat P Normative data for Trail Making Test-Thai Modification J Thai Rehabili Med 2007 17 26 30

[B25] Wechsler D WMS-III Administration and Scoring Manual 1997 The Psychological Corporation, San Antonio, TX

[B26] Thawicachat N Worakul P Kanchananakintr P Alzheimer’s disease assessment scale (ADAS) Thai version J Thai Gerontol Geriatr Med 2002 3 21 32

[B27] Harper A Power M the WHOQOL group Development of the World Health Organization WHOBREF Quality of Life Assessment Psychol Med 1998 28 551 558 9626712 10.1017/s0033291798006667

[B28] American Psychiatric Association Diagnostic and Statistical Manual of Mental Disorders 1994 4 American Psychiatric Association, Washington, DC

[B29] Petersen RC Doody R Kurz A Mohs RC Morris JC Rabins PV Current concepts in mild cognitive impairment Arch Neurol 2001 58 1985 1992 10.1001/archneur.58.12.1985 11735772

[B30] Mathias S Nayak US Isaacs B Balance in elderly patients: the “get-up and go” test Arch Phys Med Rehabil 1986 67 387 389 3487300

[B31] O’Bryant S Waring SC Cullum M Hall J Lacritz L Massman PJ Lupo PJ Staging dementia using clinical dementia rating scale sum of boxes scores Arch Neurol 2008 65 1091 1095 10.1001/archneur.65.8.1091 18695059 PMC3409562

[B32] Scheltens P Leys D Barkhof F Huglo D Weinstein HC Vermersch P Kuiper M Steinling M Wolters EC Valk J Atrophy of medial temporal lobes on MRI in “probable” Alzheimer’s disease and normal ageing: diagnostic value and neuropsychological correlates J Neurol Neurosurg Psychiatry 1992 55 967 972 10.1136/jnnp.55.10.967 1431963 PMC1015202

[B33] Fazekas F Chawluk JB Alavi A Hurtig HI Zimmerman RA MR signal abnormalities at 1.5 T in Alzheimer’s dementia and normal aging AJR Am J Roentgenol 1987 149 351 356 3496763 10.2214/ajr.149.2.351

[B34] Scheltens P Barkhof F Leys D Pruvo JP Nauta JJ Vermersch P Steinling M Valk J A semiquantative rating scale for the assessment of signal hyperintensities on magnetic resonance imaging J Neurol Sci 1993 114 7 12 10.1016/0022-510X(93)90041-V 8433101

[B35] Pasquier F Leys D Weerts JG Mounier-Vehier F Barkhof F Scheltens P Inter- and intraobserver reproducibility of cerebral atrophy assessment on MRI scans with hemispheric infarcts Eur Neurol 1996 36 268 272 10.1159/000117270 8864706

[B36] Nutt JG Marsden CD Thompson PD Human walking and higher-level gait disorders, particularly in the elderly Neurology 1993 43 268 279 10.1212/WNL.43.2.268 8437689

[B37] Abdulraheem IS Oladipo AR Amodu MO Prevalence and correlates of physical disability and functional limitation among elderly rural population in Nigeria Journal of Aging Research 2011 13 article ID 369894doi:10.4061/2011/369894 10.4061/2011/369894 PMC3124875 21748005

[B38] Barnes DE Yaffe K The projected effect of risk factor reduction on Alzheimer disease prevalence Lancet Neurol 2011 10 819 828 10.1016/S1474-4422(11)70072-2 21775213 PMC3647614

[B39] Pantoni L Basile AM Pracucci G Asplund K Bogousslavsky J Chabriat H Impact of age-related cerebral white matter changes on the transition to disability – The LADIS study: rationale Design and Methodology. Neuroepidemiology 2005 24 51 62 10.1159/000081050 15459510

[B40] Senanarong V Harphadungkit K Lertrit P Mitrpant C Udompunthyrak S Limwong C Experience of ApoE study in Thai elderly J Med Assoc Thai 2001 84 182 187 11336076

[B41] Verghese J Lipton RB Hall CB Kuslansky G Katz MJ Buschke H Abnormality of gait as a predictor of non-Alzheimer's dementia N Engl J Med 2002 347 1761 1768 10.1056/NEJMoa020441 12456852

